# The balance between lung regulatory T cells and Th17 cells is a risk indicator for the acute exacerbation of interstitial lung disease after surgery: a case-control study

**DOI:** 10.1186/s12890-023-02362-2

**Published:** 2023-02-22

**Authors:** Mariko Fukui, Norihiro Harada, Kazuya Takamochi, Takuo Hayashi, Takeshi Matsunaga, Aritoshi Hattori, Izumi Kawagoe, Kenji Suzuki

**Affiliations:** 1 Department of General Thoracic Surgery, Juntendo University School of Medicine, 1-3, Hondo 3-chome, Bunkyo-ku, 113-8431 Tokyo, Japan; 2 Respiratory Medicine, Juntendo University School of Medicine, Tokyo, Japan; 3 Human Pathology, Juntendo University School of Medicine, Tokyo, Japan; 4 Anesthesiology, Juntendo University School of Medicine, Tokyo, Japan

**Keywords:** Surgery, Acute exacerbation, Idiopathic interstitial pneumonia, Regulatory T cell, Th17

## Abstract

**Background:**

Acute exacerbation (AE) of interstitial lung disease (ILD) (AE-ILD) is a life-threatening condition and the leading cause of 30-day mortality among patients who underwent pulmonary resection for lung cancer in Japan. This study was conducted to clarify the characteristics of the immune environment of lung tissues before the onset of AE-ILD.

**Methods:**

This retrospective matched case-control study compared the immune phenotypes of helper T cells in lung tissues from patients with and without AE-ILD after surgery. In total, 135 patients who underwent surgical resection for lung cancer and were pathologically diagnosed with idiopathic interstitial pneumonia (IIP) at our institute between 2009 and 2018 were enrolled. Thirteen patients with AE-IIP and 122 patients without AE (non-AE) were matched using a propensity score analysis, and 12 cases in each group were compared. We evaluated the percentages of T helper (Th)1, Th2, Th17, regulatory T (Treg), and CD8 cells in CD3^+^ T cells and the Th1:Th2, Th17:Treg, and CD8:Treg ratios in patients with AE by immunostaining of lung tissues in the non-tumor area.

**Results:**

We found a significant difference in the lung Th17:Treg ratio between the AE and non-AE groups (1.47 and 0.79, p = 0.041). However, we detected no significant differences in the percentages of lung Th1 (21.3% and 29.0%), Th2 (34.2% and 42.7%), Th17 (22.3% and 21.6%), Treg (19.6% and 29.1%), and CD8^+^ T cells (47.2% and 42.2%) of CD3^+^ T cells between the AE and non-AE groups.

**Conclusion:**

The ratio of Th17:Treg cells in lung tissues was higher in participants in the AE group than in those in the non-AE group.

**Clinical Trial Registration:**

This study was approved by the ethics committee of our institute (2,016,095).

**Supplementary Information:**

The online version contains supplementary material available at 10.1186/s12890-023-02362-2.

## Background

Acute exacerbation (AE) of interstitial lung disease (ILD) is a lethal respiratory deterioration resulting from an unidentified cause [1–3]. Idiopathic pulmonary fibrosis (IPF) is the most common form of ILD (approximately 20–50%) [4], and up to 46% of deaths in IPF are preceded by an AE. The median survival of patients with IPF who experience AE is approximately 3 − 4 months [2].

AE of ILD (AE-ILD) is also the main cause of 30-day mortality after surgery in patients with lung cancer [5–7]. A multi-institutional retrospective study conducted by the Japanese Association for Chest Surgery showed that AE-ILD occurred in 164 (9.3%) of 1763 postoperative patients with ILD and resulted in a mortality rate of 43.9% [8]. Several reports have indicated the risk factors for AE-ILD; [8, 9] however, prediction using clinical factors is difficult. Some patients with similar, previously reported risk factors, such as male sex, low % volume capacity (VC), low % diffusing capacity of the lung for carbon monoxide (DLCO), and usual interstitial pneumonia (UIP) appearance [8, 9], have AE, while others do not. Therefore, we speculated whether the risk of AE could be determined by assessing differences in the immune environment of the lungs.

The upregulation of macrophage activation pathways is involved in lung inflammation during AE of idiopathic pulmonary fibrosis (AE-IPF). The M1 pathway is similar to the pathology of acute respiratory distress syndrome (ARDS) [10], leading to increased expression of interleukin (IL)-8 and CXCL1 and increased neutrophil recruitment via the CXCR2 receptor. The M2 pathway is a characteristic of AE-ILD, is activated by type II alveolar epithelial cell damage, and is involved in fibrosis [11] .The rapidly deteriorating cytokine profile of patients with IPF during AE-IPF has been reported to be closer to the cytokine profile of ARDS, which is predominantly characterized by inflammation (i.e., the M1 pathway) rather than accelerating the endogenous fibrosis process (i.e., the M2 pathway) [12]. Recent studies have revealed that lung inflammation mediated by CD4^+^ T cells may contribute to ARDS pathogenesis [13, 14]. However, there have been no reports regarding the balance between subgroups of T cells in the human alveolar tissue before the onset of ARDS or AE-idiopathic interstitial pneumonia (IIP).

Thus, the present study was performed to conduct a lung T cell subset analysis to investigate whether the balance of T cell subsets prior to the onset of the disease is associated with postoperative AE-IIP.

## Methods

### Patients and study design

This was a single-center, retrospective, matched case-control study that included patients with non-small cell lung cancer with IIP who underwent pulmonary resection at Juntendo University between January 2009 and June 2018. The data presented here are from an observational database of surgical candidates at Juntendo Hospital.

This study was approved by the ethics committee of Juntendo University School of Medicine (No. 2,016,095) and performed in accordance with the guidelines of the Declaration of Helsinki and its subsequent amendments. Informed consent was obtained in the form of an opt-out method on the website. This study was supported by JSPS KAKENHI (grant number 21K16523).

### Definitions

#### IIP

In this study, 137 patients were pathologically diagnosed with IIP using lung resection for lung cancer. Autoimmune-ILD and exposure-related-ILD were excluded because these diseases have different immunological backgrounds and different effects on AE. All specimens were fixed overnight in 15% buffered formalin and sectioned at 5–10 mm parallel intervals, including the tissues in areas where lung cancer was absent. Pathological IIP cases were diagnosed according to the 2002 American Thoracic Society/European Respiratory Society (ATS/ERS) guidelines [8, 14]. A diagnosis of UIP was predicated for a combination of the following: (1) patchy dense fibrotic tissues with architectural distortion; (2) a predilection for subpleural and paraseptal lung parenchyma; (3) fibroblast foci; and (4) the absence of features that suggest an alternative diagnosis.The histologic findings of other IIP were also assessed for diagnosis according to the 2002 ATS/ERS international multidisciplinary consensus guidelines [15]. Cases that could not be classified for various reasons, including inadequate clinical or radiologic information and major discrepancies between the clinical radiologic and pathologic findings, were diagnosed as unclassifiable interstitial pneumonia cases.

Clinically diagnosed IIP cases where the IIP site was not resected or where the pathological diagnosis was not confirmed were excluded from this study.

#### AE

The definition of AE-IIP in this study was based on criteria reported in 2011 [9] and 2016 [2]. AE-IIP was defined as worsening of dyspnea and hypoxia from baseline within 30 days postoperatively, including new ground-glass abnormalities and/or consolidation superimposed on a background reticular or honeycombing pattern. Findings with alternative causes, such as left heart failure, aspiration pneumonia, pulmonary embolism, or an identified cause of acute lung injury, were excluded from AE-IIP.

### Control patients

In total, 135 patients were pathologically diagnosed with IIP. Patients were divided into the AE (n = 13, 9.6%) and non-AE (n = 122, 90.4%) groups. Thirteen cases of AE-IIP and 122 cases of non-AE were matched using a propensity score analysis, and 12 cases in each group were compared.

### Immunohistochemical analysis

The subpleural lung tissues without cancer were used in this study, which was collected for the diagnosis of ILD and stored in a paraffin block. We evaluated immunohistochemical staining for CD3 and dual staining for CD4 and CD8 and also for CD4 and dual staining for T-bet, GATA3, STAT3, FOXP3, and CD8 in all non-cancer specimens.

After deparaffinization, monoclonal antibodies against CD3, CD4, CD8, T-bet, and GATA3 were added to the autostaining device. Immunohistochemical staining was performed using a commercially available autostaining device (VENTANA BenchMark GX; Roche Diagnostics K.K., Tokyo, Japan), according to the manufacturer’s protocol. Monoclonal antibodies against STAT3 (Lsbio, Seattle, WA, USA) and FOXP3 (Abcam, Waltham, MA, USA) were used for staining. A dual-labeled cell was defined by the presence of cytoplasmic staining for CD4 (red chromogen), CD3, T-bet, GATA3, STAT3, FOXP3, and CD8 (brown chromogen) and nuclear staining for the interrogated transcription factor (blue chromogen). Slides were visualized using an Olympus AX73 (Olympus Corporation, Tokyo, Japan) microscope. The dual-labeled CD3^+^/CD4^+^, CD4^+^/T-bet^+^, CD4^+^/GATA3^+^, CD4^+^/STAT3^+^, CD4^+^/ FOXP3^+^, and CD8^+^ cells were counted in over three independent areas, with the greatest abundance of lymphocytes (e-Fig. 1). T helper (Th)1 cells, Th2 cells, Th17 cells, and Tregs were identified as CD4^+^ T-bet^+^ cells, CD4^+^ GATA3^+^ cells, CD4^+^ STAT3^+^ cells, and CD4^+^ FOXP3^+^ cells, respectively. Any visible pigment, including weak or pale staining, indicated positive staining. Cell counts were performed using Image J (NIH, Bethesda, ML, USA). MF, who was blinded to the clinical backgrounds and courses of the patients, performed the immunohistochemical evaluation, and the results were later checked against clinical data. If the difference from other data was > 20%, the patient was re-evaluated. The mean number of cells counted per high-power field was used to calculate the percentages of Th1, Th2, Th17, Treg, and CD8^+^ cells.

### Statistical analysis

Clinicopathological features of patients with ILD with or without AE after surgery were investigated. Continuous variables were compared using Student’s *t*-test, Welch’s t-test, and the Mann − Whitney U test. Categorical variables were compared using the chi-squared test and Fisher’s exact test. We used a propensity score matching approach to control for clinical selection bias in the two groups when comparing the AE and non-AE immune samples. The propensity score was calculated based on the distribution of IIP, UIP pattern, preoperative arterial oxygen partial pressure, %VC, %DLCO, and the surgical procedure as independent variables. Patients were matched 1:1 using nearest-neighbor matching (caliper width:2 times the standard deviation of the propensity score on the logit scale) without replacement.

All statistical analyses were performed using IBM SPSS Statistics for Windows version 25.0 (IBM Corp., Armonk, NY, USA). P-values < 0.05 were considered statistically significant.

## Results

Clinicopathological characteristics of the matched groups are presented in Table 1. There were no statistical differences in the clinical factors between the two groups before matching (e-Table 1), and the differences between the groups decreased after matching (Table 1). More than 80% of all cases were of male individuals. There were two never-smokers in the non-AE group. Lobectomy was the most common surgical procedure. There were no cases of steroid or antifibrotic drug intake before surgery. The time to postoperative onset in the AE group was 7.5 days (2–19 days).

**Table 1:** Patient characteristics after propensity-score matching

Variable			
	AE	Non-AE	p-value
Number	12	12	
Male (%)	10 (83.3%)	10 (83.3%)	1.000
Age	73.0 ± 6.3	73.1 ± 8.9	0.978
Smoking status (pack-year)	46.2 ± 20.1	46.5 ± 41.1	0.985
KL-6 (U/ml)	734.0 ± 552.9	468.2 ± 200.9	0.162
Diffuse and central distribution of IIP in CT	3 (25.0%)	2 (16.7%)	0.611
%VC < 80%	3 (25.0%)	2 (16.7%)	0.611
FEV1/FVC < 70%	3 (25.0%)	4 (33.3%)	0.639
%DLCO	4 (33.3%)	2 (16.7%)	0.338
Preoperative pO_2_ < 70 mmHg	1 (8.3%)	2 (16.7%)	0.534
Surgical procedure			1.000
Pneumonectomy	1 (8.3%)	1 (8.3%)	
Lobectomy	8 (66.7%)	8 (66.7%)	
Segmentectomy/ wide wedge resection	2 (16.7%)	2 (16.7%)	
Histology of lung cancer (sq)	3 (25.0%)	5 (41.7%)	0.375
p-stage I	4 (33.3%)	2 (16.7%)	0.407
Pathological UIP	6 (50.0%)	6 (50.0%)	1.000
Date of AE (POD)	7.5 (2–19)		

Data are presented as the mean ± standard deviation unless otherwise indicated. AE, acute exacerbation; IIP, idiopathic interstitial pneumonia; CT, computed tomography; VC, volume capacity; FEV1.0, forced expiratory volume in 1 s; FVC, forced vital capacity; DLCO, diffusing capacity of the lung for carbon monoxide; UIP, usual interstitial pneumonia; POD, postoperative day

**Fig. 1:**
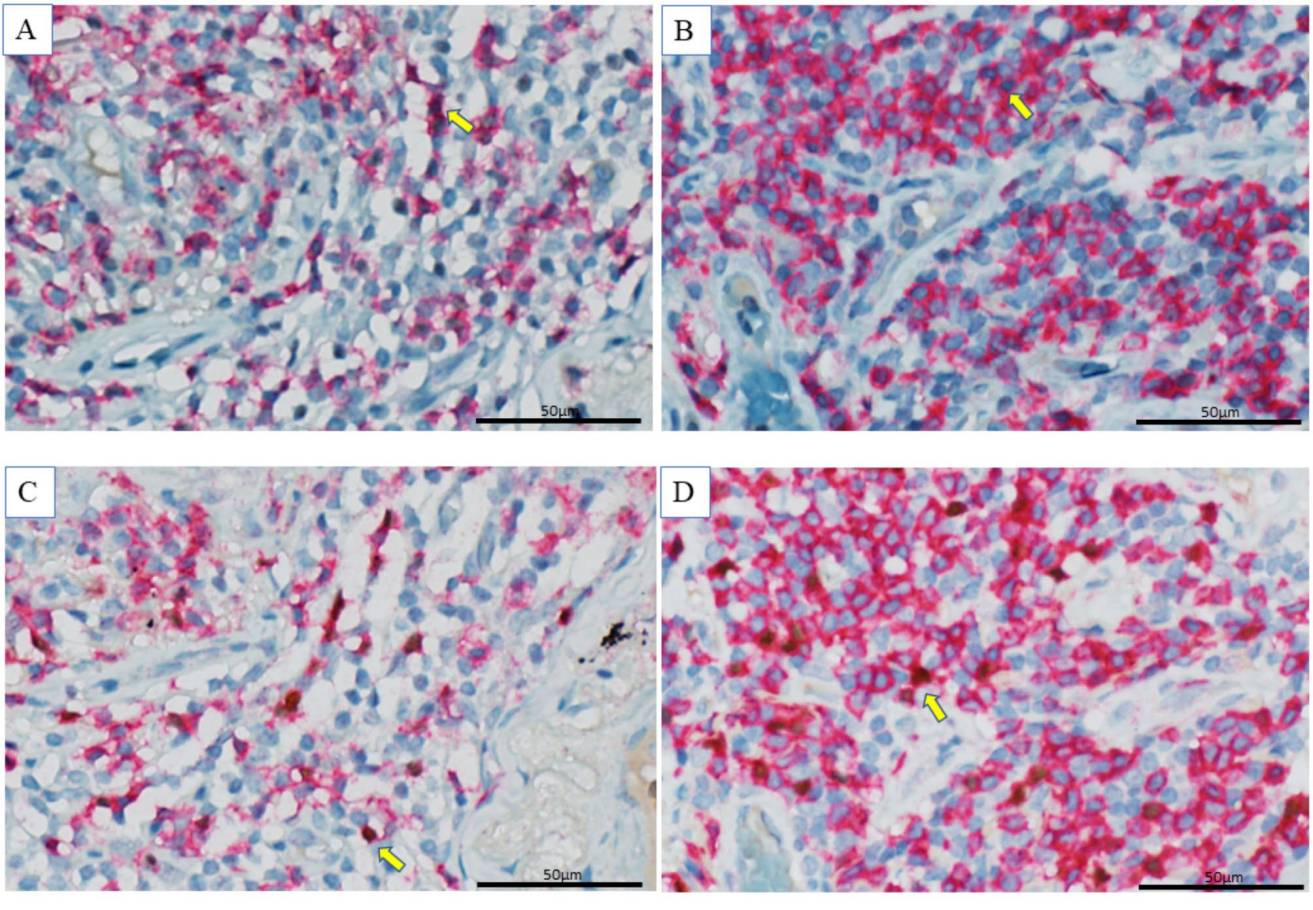
Typical immunohistochemical findings of Th17 and Treg in patients with and without AE. A dual-labeled cell was defined by the presence of cytoplasmic staining for CD4 (red chromogen), STAT3, and FOXP3 (brown chromogen) and nuclear staining for the interrogated transcription factor (blue chromogen). The yellow arrow shows an example of a double-stained cell. CD4 was more extensively stained in patients without AE. CD4^+^/STAT3^+^ were more prominent in the AE group (A) than in the non-AE group (B). CD4^+^/FOXP3 appeared less stained in the AE group (C) than in the non-AE group (D)

Figure 1 shows a typical lung section of postoperative AE-ILD (Fig. 1A and C) and ILD without AE (Fig. 1B and D). Dual-labeled cells (CD4^+^/STAT3^+^) were more prominent in patients with AE (Fig. 1A) than in those without (Fig. 1B), but dual-labeled cells (CD4^+^/FOXP3) appeared less in patients with AE (Fig. 1C) than in those without (Fig. 1D).

Table 2 presents immunohistochemical findings in the lung tissues from the postoperative AE and non-AE groups. There was a significant difference in the lung Th17:Treg ratio between the AE and non-AE groups (p = 0.041). However, we detected no significant differences between the AE and non-AE groups in the percentages of lung Th1 (21.3% and 29.0%), Th2 (34.2% and 42.7%), Th17 (22.3% and 21.6%), Treg (19.6% and 29.1%), and CD8^+^ T cells (47.2% and 42.2%) of CD3^+^ T cells. There was no difference in the lung Th17:Treg ratio between the UIP and non-UIP groups, and the ratio of lung Th2 cells was predominant in UIP (e-Table 2).

**Table 2:** Immunohistochemical findings in the AE and non-AE groups

	AE	Non-AE	p-value
Th1:Th2 ratio	0.66 ± 0.21	0.69 ± 0.17	0.755
Th17:Treg ratio	1.47 ± 0.92	0.79 ± 0.34	0.041
CD8:Treg ratio	3.64 ± 2.80	1.72 ± 0.92	0.052
Th cell subtype in CD3-positive T cells, %			
Th1	21.3 ± 10.5	29.0 ± 7.1	0.067
Th2	34.2 ± 18.1	42.7 ± 9.2	0.193
Th17	22.3 ± 8.6	21.6 ± 8.0	0.858
Treg	19.6 ± 12.9	29.1 ± 12.3	0.101
CD8	47.2 ± 15.4	42.2 ± 15.4	0.409

Data are presented as the mean ± standard deviation. AE, acute exacerbation; Th, T helper cell; Treg, regulatory T cell

## Discussion

To our knowledge, this is the first study to show that the ratio of Th17 to Treg cells in the lung tissues of patients with ILD and postoperative AE was higher than that in those without AE.

There are few reports regarding the AE-ILD and cytokines, but Senoo et al. [16] reported a relationship between AE-IPF and the cytokine levels. They reported that the levels of interleukin (IL)-17 and IL-23 in bronchoalveolar lavage fluid of patients with AE-IPF increased, and Th17 cells were the predominant source of IL-17 A in mice with AE of IPF [16]. Moye et al. also reported the relationship between T cells and AE-IPF; Treg depletion in wild-type mice with established lung fibrosis significantly worsened infection-induced fibrosis exacerbation, and Treg expansion completely inhibited AE [17]. In the present study, the percentage of lung Treg and Th17 cells among T cells was not significantly different between the postoperative AE and non-AE groups, but the ratio of the lung Th17 to Treg cells was higher in the AE group, supporting previous findings in mouse models that Tregs suppress the onset of AE and that Th17 promotes the onset of AE.

There have been numerous reports regarding the relationship between ARDS pathogenesis and cytokine levels. Imbalances in Th17 or other inflammatory subsets in ARDS promote an increase in autoimmune disease. IL-17 acts synergistically with IL-6, tissue necrotic factor, and rapid neutrophils [18]. With the elucidation of the involvement of cytokines, clinical trials using IL-6 antagonists for ARDS caused by coronavirus disease have been established clinically. The utility of IL-6 antagonists has been reported in a meta-analysis [19]. Although AE-ILD is a fatal and clinically important problem, its pathophysiology is difficult to elucidate because it occurs less frequently than ARDS. In this study, the involvement of lung Th17 cells before the onset of postoperative AE-ILD was suggested; this may be useful for future pathological elucidation and therapeutic research.

The present study has some limitations. First, this was a retrospective study conducted at a single center. Therefore, this study may lack external validity. We would like to verify whether the results can be demonstrated using more cases. Second, all specimens used in this study were of patients with lung cancer. Several studies have shown that the composition of Tregs and Th17 cells may be altered in the tumor microenvironment, and that these two CD4 + T cell subsets play active roles in promoting lung cancer progression and metastasis [20]. The presence of lung cancer may have influenced the distribution of T cells in the background lung. Third, the lung parenchyma to be evaluated was defined in this study. This is a limitation because there were only a few residual specimens of non-tumor tissues. The specimens were of subpleural lung parenchyma with typical ILD findings. AE-ILD often develops from the subpleural lung parenchyma, regardless of laterality or lobe; therefore, we believe that the specimens in this study are useful as an evaluation site. However, the distribution of T cells in the lungs may vary depending on the section being evaluated. Further prospective studies are required to confirm our findings.

## Conclusion

There may be a difference in the balance of T cell subgroups in lung tissues before the onset of AE after surgery. The ratio of lung Th17 to Treg cells was higher in the AE group than in the non-AE group.

## Electronic supplementary material

Below is the link to the electronic supplementary material.

Supplementary Material 1

Supplementary Material 2

## Data Availability

The data are available from the corresponding author on reasonable request. The data are not publicly available owing to privacy reasons.

## List of abbreviations

AE: Acute exacerbation
AE-ILD: Acute exacerbation of interstitial lung disease
AE-IPF: Acute exacerbation of idiopathic pulmonary fibrosis
ARDS: acute respiratory distress syndrome
CD: cluster of differentiation
DLCO: diffusing capacity of the lung for carbon monoxide
IIP: idiopathic interstitial pneumonia
IL: interleukin
ILD: interstitial lung disease
IPF: idiopathic pulmonary fibrosis
PS: propensity score
Th: T helper
Treg: regulatory T cell
UIP: usual interstitial pneumonia
VC: volume capacity
