# Supplementary figures and images for: The balance between lung regulatory T cells and Th17 cells is a risk indicator for the acute exacerbation of interstitial lung disease after surgery: a case-control study
Source: BMC Pulm Med. 2023 Feb 22;23:70. doi: 10.1186/s12890-023-02362-2 (PMC9945823; doi:10.1186/s12890-023-02362-2)

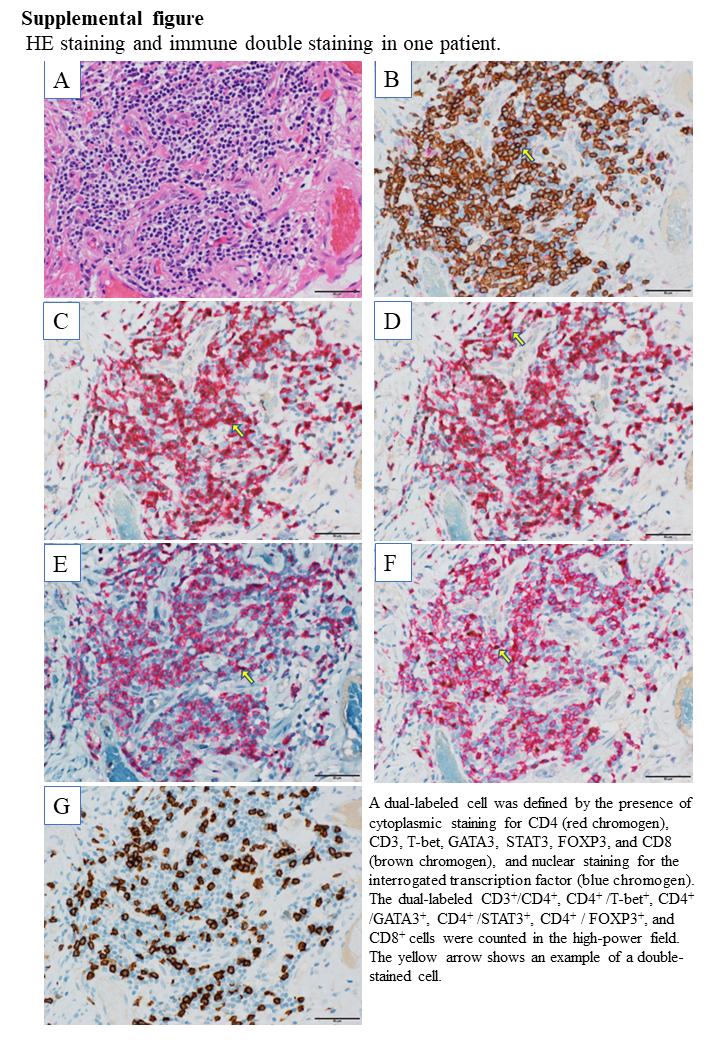

Supplement: Supplementary file 1 — Supplementary Material 1 [file 12890_2023_2362_MOESM1_ESM.tif]
