# Supplementary material for: The balance between lung regulatory T cells and Th17 cells is a risk indicator for the acute exacerbation of interstitial lung disease after surgery: a case-control study
Source: BMC Pulm Med. 2023 Feb 22;23:70. doi: 10.1186/s12890-023-02362-2 (PMC9945823; doi:10.1186/s12890-023-02362-2)
Supplement: Supplementary file 2 — Supplementary Material 2 [file 12890_2023_2362_MOESM2_ESM.docx]

e-Table 1. Patient characteristics before propensity score matching.

| Variable |  | | |
| --- | --- | --- | --- |
|  | AE | Non-AE | p-value |
| Number | 13 | 122 |  |
| Male (%) | 13 (100%) | 102 (83.6%) | 0.144 |
| Age | 72.1 ± 6.3 | 72.0 ± 6.2 | 0.954 |
| Smoking status | 56.9 ± 42.1 | 50.4 ± 28.1 | 0.467 |
| Diffuse and central distribution of IIP in CT | 3 (23.1%) | 27 (22.1%) | 0938 |
| %VC < 80% | 3 (23.1%) | 26 (21.3%) | 0.767 |
| FEV1.0/FVC <70% | 3 (23.1%) | 29 (23.7%) | 0.936 |
| %DLCO <40% | 4 (30.8%) | 55 (45.1%) | 0.242 |
| Preoperative pO2 <70 mmHg | 2 (15.4%) | 11 (9.0%) | 0.403 |
| Surgical procedure |  |  | 0.606 |
| Pneumonectomy | 1 (7.7%) | 2 (1.6%) |  |
| Lobectomy | 6 (46.2%) | 103 (84.4%) |  |
| Segmentectomy/ wide wedge resection | 2 (15.4%) | 13 (10.7%) |  |
| Histology of lung cancer (sq) | 4 (30.8%) | 63 (51.6%) | 0.153 |
| p-stage I | 8 (61.5) | 57 (46.7%) | 0.365 |
| Pathological UIP | 7 (53.8%) | 53 (43.4%) | 0.473 |
| Other IIP | 5 (46.2%) | 69 (66.6%) |  |
| Non-specific interstitial pneumonia | 1 | 2 |  |
| Unclassifiable | 5 | 42 |  |

Data are presented as the mean ± standard deviation.

e-Table 2. Immunohistochemical findings in UIP and non-UIP patients

|  | UIP (n = 14) | Non-UIP (n = 10) | p-value |
| --- | --- | --- | --- |
| Th1: Th2 ratio | 0.61 ± 0.15 | 0.75 ± 0.21 | 0.092 |
| Th17: Treg ratio | 1.33 ± 1.02 | 0.98± 0.43 | 0.108 |
| CD8: Treg ratio | 1.91 ± 1.06 | 3.63 ± 2.95 | 0.337 |
| Th cell subtype in CD3 positive T cells, % | | | |
| Th1 | 27.2 ± 8.9 | 22.5 ± 10.3 | 0.279 |
| Th2 | 45.6 ± 14.7 | 30.1 ± 10.5 | 0.013 |
| Th17 | 24.3 ± 8.8 | 19.4 ± 8.5 | 0.172 |
| Treg | 24.3 ± 7.4 | 19.1 ± 9.3 | 0.095 |
| CD8 | 42.1 ± 12.1 | 47.8 ±14.9 | 0.909 |

Data are presented as the mean ± standard deviation.
